# Improving Risk Stratification and Surgical Decision-Making for Unruptured Cerebral Aneurysm: A Proof-of-Concept of Novel Morphological Parameters and Computational Hemodynamics

**DOI:** 10.3390/brainsci16090970

**Published:** 2026-09-14

**Authors:** Giuseppe Roberto Giammalva, Gabriele Costantino, Umberto Emanuele Benigno, Lapo Bonosi, Micol Tantillo, Antonino Cirello, Vito Ricotta, Tommaso Ingrassia, Giuseppe Craparo, Giovanni Tringali

**Affiliations:** 1 Unit of Neurosurgery, ARNAS Civico Di Cristina Benfratelli, 90127 Palermo, Italy; 2 Department of Engineering, University of Palermo, 90100 Palermo, Italy; 3 Unit of Neuroradiology, ARNAS Civico Di Cristina Benfratelli, 90127 Palermo, Italy

**Keywords:** unruptured intracranial aneurysm, computational fluid dynamics, aneurysm operability, surgical strategy, integrative approach

## Abstract

**Highlights:**

**What are the main findings?**

- A newly derived aneurysm morphological parameter in unruptured aneurysms shows a strong association with subsequent rupture risk, identifying adverse shapes at higher risk.
- These adverse morphologies exhibit distinct, quantifiable hemodynamic patterns, including elevated intra-aneurysmal pressure and abnormal wall shear stress.

**What are the implications of the main findings?**

- Morphological–hemodynamic metrics provide objective, reproducible parameters that can support neurosurgical decision-making in selecting aneurysms for treatment versus conservative follow-up.
- Integrating this quantitative framework into preoperative assessment may reduce reliance on subjective judgment, refine surgical planning, and ultimately enable safer, more individualized intracranial aneurysm management.

**Abstract:**

Background: Unruptured intracranial aneurysms (UIAs) represent a major challenge in neurosurgical practice. Rupture risk assessment traditionally relies on qualitative parameters, such as aneurysm size and shape, combined with surgical judgment and clinical expertise. However, these approaches may fail to account for the complex interplay between vascular morphology and hemodynamic forces that leads to aneurysm growth and rupture. Therefore, the development of reliable and quantitative parameters is essential to support neurosurgeons in balancing the risks of intervention against the natural history of the disease, thus optimizing decision-making for surgery. Methods: We developed an integrative framework based on a novel morphological parameter (MP), integrating multiple geometric descriptors into a single quantifiable index. To complement morphological assessment, patient-specific computational fluid dynamics (CFD) simulations were performed to evaluate hemodynamic factors known to influence aneurysm progression, thus analyzing structural and flow-related determinants of rupture risk. Results: In a cohort of 60 consecutive patients with MCA UIAs, MP showed strong concordance with the treatment decision made by an expert neurosurgeon regarding the indication for surgical clipping of aneurysms considered at risk of imminent rupture, providing an indirect, objective measure of UIA rupture risk stratification. CFD analyses confirmed that adverse hemodynamic conditions, including elevated intra-aneurysmal pressures and heterogeneous distribution of wall shear stress, correlated with unfavorable morphological features that were considered to indicate a higher risk of rupture by an expert neurosurgeon. Moreover, the integration of expert clinical judgment with quantitative indices may enhance the discrimination between aneurysms requiring early surgical intervention and those suitable for conservative management. Conclusions: This proof-of-concept study highlights the potential added value of combining morphological modelling and patient-specific hemodynamic analysis to support, rather than replace, expert neurosurgical judgment regarding the surgical management of unruptured MCA aneurysms. The proposed parameter (MP) shows concordance with expert operability decisions and may complement current neurosurgical evaluation as an ancillary decision-support tool, potentially contributing to safer operative strategies, more individualized patient management, and ultimately improved patient outcomes in the treatment of unruptured intracranial aneurysms.

## 1. Introduction

Cerebral aneurysms are pathological and localized wall dilatations of cerebral arteries. Their pathogenesis is multifactorial and still incompletely understood, likely involving a complex interplay of genetic predisposition and molecular alterations [1,2]. Subarachnoid hemorrhage from aneurysmal rupture accounts for 7% of all strokes and represents the primary cause of non-traumatic subarachnoid hemorrhage. It represents a devastating condition, with high mortality and severe neurological sequelae among survivors, who are frequently burdened by significant disability and reduced quality of life [3]. The estimated prevalence of unruptured intracranial aneurysms (UIAs) in the general population is 3.2%, and their incidence is expected to rise because of early identification facilitated by advancements in, and broader access to, modern neuroimaging techniques.

As the incidence of UIAs continues to rise, accurate stratification of rupture risk become of pivotal importance [4]. In fact, optimal management of UIAs remains one of the most debated issues in vascular neurosurgery, and traditional risk assessment still largely relies on clinical scores such as PHASES, UIATS, and ELAPSS, which often fail to fully account for patient-specific morphology of aneurysm and its hemodynamic characteristics [5,6]. Still, treatment recommendations based on risk stratification mostly rely on the International Study of Unruptured Intracranial Aneurysm (ISUIA), despite this study being notably affected by several methodological limitations. In addition to ISUIA [7], other major studies, such as BRAT, CARAT, SAHIT, and ATENA [8,9,10,11], have attempted to refine UIA risk stratification; however, none have provided definitive conclusions or universal criteria for clinical decision-making.

Commonly, hemodynamics has been considered a key determinant in the pathogenesis, progression, and rupture of intracranial aneurysm. In this regard, recent advances in computational fluid dynamics (CFD) have provided novel insights into aneurysm wall behavior, even in cases of dome irregularity and the presence of blebs, allowing quantitative assessment of wall shear stress (WSS), intra-aneurysmal pressure, and flow instability.

In this article, we propose a translational framework that integrates CFD-derived parameters with a novel morphological index called a morphological parameter (MP), with the aim of correlating morphological and hemodynamic features of UIAs (e.g., focal wall shear stress heterogeneity, flow instability and their correlation with dome irregularities and blebs) for supporting operative decision-making by prediction of aneurysm rupture [12]. This proof-of-concept study focused on middle cerebral artery (MCA) UIAs, the most frequently treated aneurysms by surgery, which therefore pose a critical challenge in determining whether and when surgical intervention is warranted before a sudden rupture occurs.

Furthermore, this study highlights the potential role of such an integrated translational framework in preoperative surgical planning, by providing a roadmap for identifying regions at elevated risk of rupture during aneurysm dissection, thereby improving surgical safety and ultimately improving patient outcomes.

## 2. Materials and Methods

### 2.1. Patient Selection and Imaging Workflow

This study retrospectively included 60 patients (M:F ratio 1:3, mean age 57 ± 11 yr) with MCA bifurcation UIAs, who were admitted to A.R.N.A.S. Civico (Palermo—Italy) between 2023 and 2024 [Table 1]. Imaging datasets with 3D rotational angiography, CTA, and MRI, were retrieved for segmentation and computational analysis.

UIA segmentation was performed with 3D Slicer, an open-source software for segmentation and model reconstruction. The workflow included manual refinement of the vascular surface and extension of inflow and outflow boundaries to ensure stable CFD computation as previously described in detail [12].

### 2.2. Computational Modeling

After UIA segmentation, each model underwent mesh generation and fluid dynamic simulation using ANSYS CFX software (version 2024R2; Ansys, Inc.—Canonsburg, PA, USA). Blood was modeled as a non-Newtonian fluid, with viscosity decreasing as shear rate increased, and blood viscosity was described following the Carreau viscosity model. Physiologically realistic boundary conditions were applied, with inlet velocities ranging between 0.4 and 0.8 m/s based on the mean flow rate of the MCA. Both laminar and turbulent models were tested, confirming no significant hemodynamic differences, consistent with prior studies.

### 2.3. Morphological Parameter (MP)

For the purpose of our study, aneurysm width (W), neck dimensions (N) and dome height (H) from UIA segmentation on brain CTA were adopted for the calculation of a novel Morphological Parameter (MP) to be employed in daily clinical practice for evaluating the need of surgical intervention. The neck parameters were determined as the maximum longitudinal (N) and transverse (N_T_) widths of the aneurysm neck. Aneurysm width (W) was measured as the maximum transverse diameter of the dome, and dome height (H) as the maximal perpendicular distance from the neck plane to the dome apex. Building on these measurements, several dimensionless ratios were also calculated, including the height-to-width ratio (H/W), the width-to-neck ratio (W/N), and the height-to-neck ratio (H/N, also known as the aspect ratio), the latter typically describing whether an aneurysm is elongated or spherical and how it expands relative to its neck.

The Morphological Parameter (MP) combines these variables into a single formula, as previously described in detail [12]:

$$MP=\frac{W}{N}*\frac{W+N}{2}*H*N_{T}$$

This parameter provides a composite morphological score that is sensitive to UIA geometric asymmetry and neck configuration. A threshold of 0.03 on the normalized MP scale (0–1)—corresponding to a raw MP value of approximately 53–63—was arbitrarily defined based on the distribution of MP values and clinical operability judgment in the study population; as previously reported, concordance with expert opinion regarding the need for surgical intervention increases significantly for MP values equal to or above this threshold [12].

Thus, the capability of the MP to predict UIA rupture was compared with the opinion of an expert neurosurgeon based on PHASES classifications in a blinded manner, to assess its concordance with clinical score–based operative decisions.

### 2.4. Statistical Analysis

Continuous variables (age, aneurysm size, MP) were compared between groups using Welch’s *t*-test, while categorical variables (sex, hypertension, smoking status, multiple UIA) were compared using Fisher’s exact test, given the relatively small subgroup sizes. Results were considered statistically significant at *p* < 0.01.

McNemar’s test for paired binary data was used to compare the concordance of the MP, and separately of the PHASES classification, against the treatment decision made by an expert neurosurgeon (gold standard) regarding the indication for surgical intervention. For each comparison, the continuity-corrected McNemar χ^2^ statistic (1 degree of freedom) was calculated based on the discordant cells; given the small number of discordant pairs observed in some comparisons, the exact binomial McNemar test was also computed and reported as a more conservative estimate. Statistical significance was set at *p* < 0.01. The same approach was applied to directly compare the MP against the PHASES classification within the subgroup of aneurysms judged operable by the expert surgeon.

We acknowledge that deriving and testing the MP threshold within the same cohort carries a risk of inflated diagnostic performance; accordingly, in this proof-of-concept study, results should be interpreted as hypothesis-generating rather than confirmatory. Independent validation using a separate patient cohort and blinded to threshold derivation is planned in a subsequent phase of this research [12].

### 2.5. Hemodynamic Metrics

Key CFD-derived indicators included wall shear stress (WSS), intra-aneurysmal pressure (IAP), and velocity field uniformity. Regions of low WSS were mapped onto the aneurysm dome, correlating them with morphological irregularities and wall protrusions observed on 3D renderings [13,14].

## 3. Results

Among the 60 aneurysms evaluated, 38 were deemed operable according to the treatment decision made by an expert neurosurgeon, based on judged risk of imminent rupture. Within this subgroup, the MP exceeded the predefined threshold (MP ≥ 0.03) in 34 cases, showing almost perfect concordance with expert clinical judgment regarding the indication for surgical clipping (sensitivity 89.5%; specificity 100%; PPV 100%; NPV 84.6%) (Table 2). McNemar’s test confirmed a non-statistically significant discordance between the MP and expert judgment (four discordant pairs, all in the same direction: surgeon-positive/MP-negative; χ^2^ = 2.25, df = 1, *p* = 0.134, continuity-corrected; exact binomial *p* = 0.125), consistent with the very small number of discordant cases, which may plausibly reflect random variability rather than a systematic misclassification by the MP. In comparison, the PHASES score showed markedly lower sensitivity (44.7%) and lower NPV (51.2%), while maintaining similar specificity and PPV (100%) [Table 3]; the discordance between PHASES and expert judgment was highly significant (21 discordant pairs; χ^2^ = 19.05, df = 1, *p* = 1.27 × 10^−5^, continuity-corrected; exact binomial *p* < 0.001), reflecting the comparatively poor agreement of PHASES with expert operability judgment in this cohort. A direct comparison of the MP against PHASES within the Surgeon-positive subgroup (n = 38) [Table 4] confirmed a statistically significant difference between the two classifications (17 discordant pairs, all in the same direction: MP-positive/PHASES-negative; χ^2^ = 15.06, df = 1, *p* = 1.04 × 10^−4^, continuity-corrected; exact binomial *p* = 1.53 × 10^−5^), indicating that the MP identified a significantly greater proportion of aneurysms that were PHASES-negative but nonetheless judged operable by the expert surgeon as high-risk. These findings suggest that the MP shows closer concordance with expert operability judgment than PHASES in this cohort, and may serve as a complementary decision-support tool alongside expert clinical evaluation, pending external validation [12].

CFD maps consistently revealed that regions of low WSS (<0.4 Pa) corresponded to thin-walled blebs and irregular domes, often identified intraoperatively as fragile zone [13,15]. Conversely, aneurysm necks exhibited moderate WSS levels compatible with stable flow recirculation. These findings suggest that integrating MP and WSS data preoperatively can identify regions of increased rupture susceptibility, improving the safety of surgical manipulation or possibly redefining surgical strategy.

### Case Description

**Figure 1:**
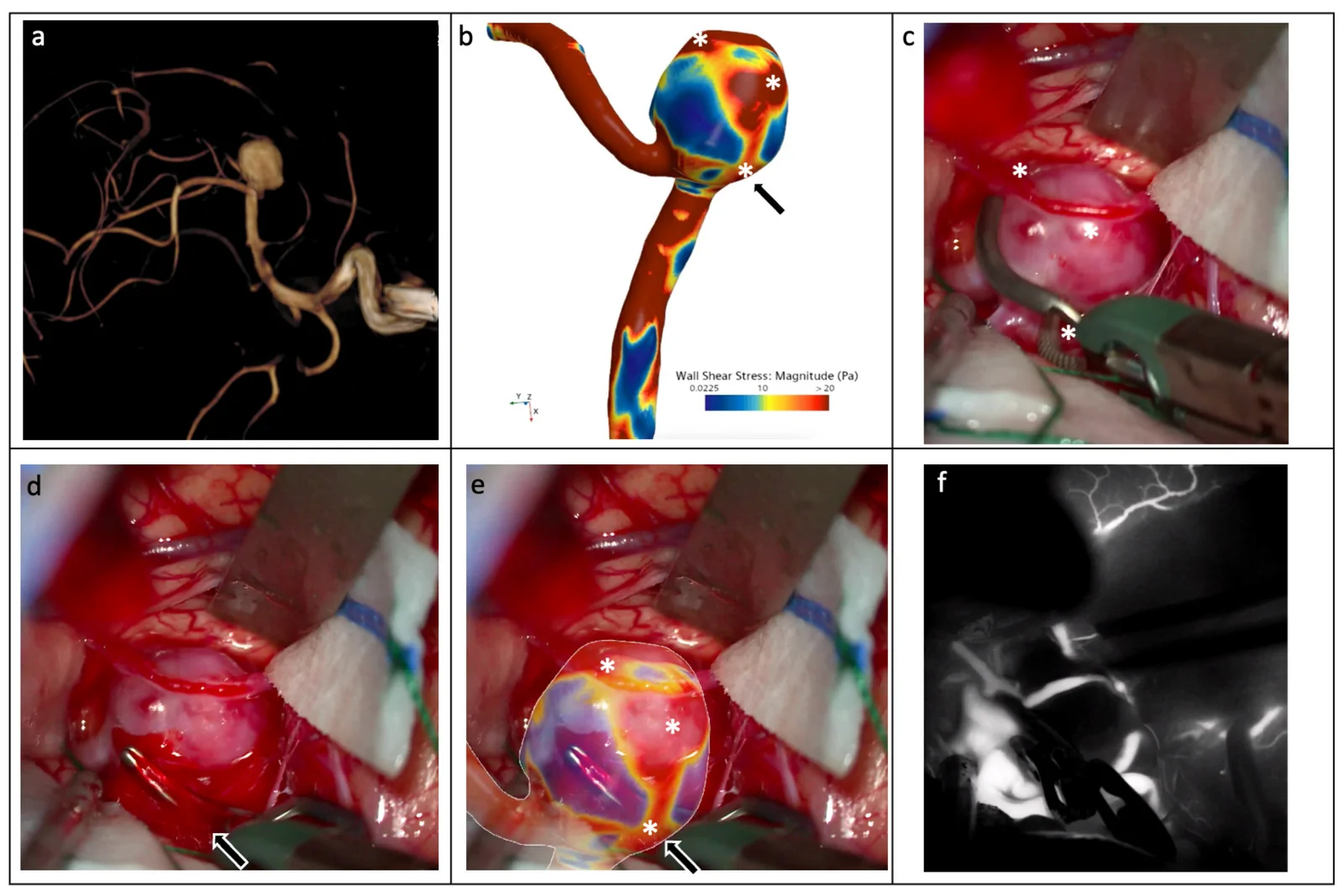
(**a**) Preoperative brain CTA showing right MCA UIA, which was further adopted for segmentation and CFD modeling. (**b**) UIA CFD model showing heterogeneous wall shear stress with focal areas of high wall pressure. (**c**) intraoperative UIA dissection, showing marked fragility of UIA dome. (**d**,**e**) intraoperative spontaneous rupture at the base of the dome, at the point of elevated wall pressure, as demonstrated by the overlay of former 3D CFD model. (**f**) successful clipping with complete exclusion of MCA aneurysm. *: areas of elevated shear stress and high wall pressure; **black arrow**: point of intraoperative spontaneous rupture.

To illustrate, in an anecdotal manner, the potential clinical relevance of the proposed morphological and hemodynamic framework, we present the case of a 47-year-old woman with a new onset of headache, who underwent CTA and MR angiography, which revealed an UIA of the right MCA at the M1–M2 bifurcation. Her medical history was notable for arterial hypertension, mild aortic and mitral valve insufficiency, and uterine leiomyomatosis treated with combined estrogen–progestin therapy. Diagnostic cerebral digital subtraction angiography (DSA) confirmed the presence of an unruptured, wide-necked (5.4 mm) saccular aneurysm of the right MCA bifurcation (5.5 × 6.8 mm), with irregular morphology characterized by wall lobulations and an apical bleb (Figure 1a). After a multidisciplinary discussion involving vascular neurosurgeons and interventional neuroradiologists, the patient was scheduled for surgical treatment.

The preoperative CTA study was analyzed according to the aforementioned protocol. After image segmentation, the UIA model underwent mesh generation and CFD analysis, which demonstrated heterogeneous wall shear stress with focal areas of elevated wall pressure up to 20 Pa, mostly at the level of the apical bleb and in proximity of the neck, and an MP value of 0.6, which was concordant to expert neurosurgical opinion and indication for surgery (Figure 1b). On this basis, the patient underwent right pterional craniotomy for microsurgical clipping of the aneurysm.

Intraoperative inspection revealed marked fragility of the aneurysmal dome (Figure 1c). During careful dissection, a spontaneous rupture occurred at the base of the dome in proximity of the neck, as predicted by the preoperative CFD findings and our computational model (Figure 1d,e). The aneurysm was successfully excluded from the circulation using two mini-clips, and the procedure was completed without further complications (Figure 1f). Postoperative CTA and DSA demonstrated complete occlusion of the aneurysm without residual filling. The postoperative course was uneventful, and the patient was discharged from the hospital on the sixth postoperative day in good clinical condition.

Despite a single case being insufficient to validate an entire model, in this illustrative case, MP and CFD findings were anecdotally concordant with the intraoperative presentation of MCA aneurysm and potential predictors of its rupture.

## 4. Discussion

The management of UIAs still poses significant surgical and clinical challenges, particularly regarding the accurate prediction of aneurysm growth and rupture risk and the optimal timing of intervention [16]. Conventional assessment methods and clinical scoring systems—largely based on aneurysm size, dome-to-neck ratio and anatomical location—are still insufficient to capture the complex biomechanical and pathophysiological factors underlying aneurysm behavior [17,18,19]. In addition, real-world studies applying PHASES, UIATS, and ELAPSS scores have shown that, while these tools assist in stratifying patients into broad risk categories, their concordance with actual clinical decision-making and long-term outcome remains only moderate, and substantial inter-observer variability persists in borderline cases. This underscores the ongoing need for complementary, patient-specific tools—such as morphological and hemodynamic metrics—that may refine decision-making beyond population-based risk scores.

Our preliminary study seeks to address this gap by introducing a novel morphological parameter (MP) which, when combined with patient-specific hemodynamic analysis, offers an objective and reproducible framework to refine risk stratification. In fact, our results demonstrate that this new MP closely aligns with expert neurosurgical assessments of operability and rupture risk, supporting its potential as a complementary tool in preoperative planning. Notably, the concordance observed between morphological characteristics and specific hemodynamic features reinforces the notion that aneurysm rupture is not governed by a single determinant, but rather by the complex interplay between vascular geometry and local flow dynamics [20,21]. A comparison of the MP with established morphological indices has been previously reported by our group, to which we refer the reader for a full methodological discussion; the present proof-of-concept study focuses specifically on evaluating the MP’s concordance with expert operability judgment.

From a neurosurgical perspective, the integration of these parameters has direct implications for clinical decision-making. In routine practice, distinguishing aneurysms that warrant immediate intervention from those that can be safely monitored is often challenging, particularly when traditional metrics are inconclusive or borderline [22,23]. An objective, quantitative measure that reflects both structural and physiological risk factors may overcome subjective interpretations and clinical scores, thereby enhancing consistency in management strategies of UIAs across surgeons and institutions.

Moreover, the framework we propose may be especially valuable in borderline cases, in which the risks associated with surgical or endovascular treatment must be carefully balanced against the expected natural history of the aneurysm. This study also highlights the translational value of integrating CFD analysis with established clinical scoring systems. While PHASES and UIATS remain indispensable for patient selection, they lack resolution regarding aneurysm wall vulnerability. The introduction of MP and related CFD-derived indices helps to bridge this gap, transforming risk estimation from a global probability into a topographic risk map that can guide both treatment indication and intraoperative strategy.

Based on the current scientific literature, our findings can be interpreted in light of three major strands of evidence linking hemodynamics to aneurysm instability: first, patient-specific CFD phenotyping, which has shown that ruptured and unruptured aneurysms exhibit distinct flow (e.g., complex inflow jets, concentrated impingement zones, and high oscillatory shear index, OSI) as reported by Cebral et al. [13]; second, the dual-mechanism hypothesis proposed by Meng et al., which suggests that aneurysm growth and rupture may be driven either by chronically high wall shear stress (WSS) with steep spatial gradients, leading to mural-cell-mediated remodeling, or by low WSS combined with high OSI, favoring inflammatory-cell-mediated wall degeneration [15]; and third, recent work on morphology–hemodynamics interactions, which underscores the importance of integrating geometric descriptors (e.g., size, aspect ratio, non-sphericity) with local shear metrics for rupture risk modeling [14].

Within this framework, the MP acts as a morphology aggregator that can be spatially mapped onto WSS and OSI distributions, thereby generating a topographic risk atlas which can be directly exploitable during microsurgical dissection. Recent histopathological studies have provided biological support for this approach, showing that arterial wall regions exposed to abnormal hemodynamic conditions, as shown by CFD analysis, exhibit endothelial dysfunction, inflammatory infiltrates, and extracellular matrix degradation, all features associated with wall thinning and fragility [24,25,26]. Integrating such tissue-level data with CFD-derived metrics reinforces the translational link between flow patterns and the microscopic substrate of aneurysm instability.

The emergence of artificial intelligence (AI) and machine learning (ML) frameworks is further transforming CFD-based risk modeling. Automated pipelines for vascular segmentation, model construction and flow simulation, as reported by Valen-Sendstad et al. [20], are enabling faster, standardized, and clinically scalable hemodynamic analysis. Coupling these AI-driven tools with morphological indices such as MP could ultimately yield a predictive, continuously learning system for real-time aneurysm assessment, bridging advanced computational modeling with everyday neurosurgical decision-making.

Ultimately, for younger neurosurgeons, CFD-derived morphological and hemodynamic maps may serve as powerful didactic and operative tools. By visualizing spatial variations in WSS and pressure before surgery, the surgeon can anticipate which regions of the aneurysm dome are most fragile and should either be avoided during microdissection or approached with extreme meticulousness, thereby complementing intraoperative microscopic cues such as wall translucency or pulsatility. Furthermore, CFD-informed visualizations integrated into neuronavigation platforms (e.g., 3D Slicer or Quicktome) can be used intraoperatively as overlays, augmenting real-time awareness of high-risk zones. Such integration has the potential to shorten the learning curve and standardize risk awareness among less experienced surgeons, promoting safer aneurysm handling and, ultimately, improved postoperative outcomes.

However, this work has several limitations. First, the sample size is relatively small and restricted to MCA aneurysms, which may limit the generalizability of the findings. Moreover, only the sensitivity and specificity of the MP and the concordance with expert judgment were tested given the limited sample size; further assessment of the accuracy and predictability of the MP with ROC analysis and multivariate modeling against established predictors of aneurysm rupture will be tested on a large and adequately powered cohort in future work.

Second, the MP threshold was derived and subsequently tested as described in a previous work within the same 60-patient cohort and expert judgment, which carries an inherent risk of overfitting and may inflate diagnostic performance metrics. However, this proof-of-concept study represents only the first stage of a broader research program comprising external and independent validation on a larger cohort.

Additionally, the MP formula, as a purely geometric index, does not explicitly incorporate radiomic descriptors of dome irregularity or the presence of blebs, both of which are recognized as decisive drivers of individual neurovascular decision-making. Within our integrated framework, however, this limitation is mitigated by the complementary CFD analysis, which captures the local hemodynamic consequences of such irregular features, as exemplified by the illustrative case reported above. Future iterations of this framework may nonetheless benefit from incorporating dedicated radiomic irregularity indices directly into the morphological component, further strengthening the synergy between geometric and hemodynamic assessment.

Third, CFD simulations remain computationally demanding and sensitive to segmentation quality, underscoring the need for standardized, reproducible image-processing and modeling pipelines. Fourth, large-scale clinical validation is still lacking, and real-world variability in CFD outputs across centers and workflows remains a concern. Nevertheless, as computational resources and semi-automated or fully automated pipelines become increasingly accessible, the integration of CFD into routine neurosurgical practice appears progressively more feasible and may ultimately contribute to more precise and reproducible aneurysm risk assessment.

## 5. Conclusions

This proof-of-concept study suggest that the combined use of a novel morphological index with hemodynamic analysis has the potential to refine current paradigms of aneurysm risk assessment, supporting expert clinical evaluation. In particular, integrating MP and CFD-derived metrics in the evaluation of unruptured intracranial aneurysms according to their particular irregularities and local fluid dynamics may support a more granular and patient-specific estimation of rupture risk than is achievable with conventional scoring systems alone or expert judgment. Future studies involving external validation, larger patient cohorts and longitudinal follow-up will be essential to prospectively validate these parameters, but the present results represent a promising step toward more individualized and objective neurosurgical management of intracranial aneurysms.

## Figures and Tables

**Table 1:** Cohort baseline characteristics of MCA UIAs patients.

	Surgery Indicated (n = 38)	Surgery Not Indicated (n = 22)	*p* Value
Age, mean ± SD	58.37 ± 10.98	51.91 ± 9.99	0.024
Sex (M/F)	7/38	5/22	0.947
Aneurysm size (mm)	7.54 ± 3.03 mm	2.93 ± 0.97 mm	**<0.001**
MP, mean ± SD	272.5 ± 313.1	19.9 ± 15.2	**<0.001**
Hypertension, n (%)	15/38	10/22	0.856
Smoking, n (%)	21/38	10/22	0.642
Multiple UIA, n (%)	10/38	4/22	0.688

**Table 2:** Comparison between MP and opinion of expert surgeon in identifying UIAs at high risk of rupture requiring surgical intervention.

	MP +	MP −	Total
Surgeon +	34	4	38
Surgeon −	0	22	22
Total	34	26	60

McNemar’s χ^2^ = 2.25, df = 1, *p* = 0.134; exact binomial *p* = 0.125.

**Table 3:** Comparison between PHASES score and opinion of expert surgeon in identifying UIAs at high risk of rupture requiring surgical intervention.

	PHASES +	PHASES −	Total
Surgeon +	17	21	38
Surgeon −	0	22	22
Total	17	43	60

McNemar’s χ^2^ = 19.05, df = 1, *p* < 0.001; exact binomial *p* < 0.001.

**Table 4:** Comparison between MP and PHASES score in identifying UIAs at high risk of rupture requiring surgical intervention, restricted to the subgroup of patients with a positive surgeon’s evaluation for operability (n = 38).

	PHASES +	PHASES −	Total
MP +	17	17	34
MP −	0	4	4
Total	17	21	38

McNemar’s χ^2^ = 15.06, df = 1, *p* < 0.001; exact binomial *p* < 0.001.

## Data Availability

The data presented in this study are available on request from the corresponding author due to institutional policy.

## Abbreviations

The following abbreviations are used in this manuscript:

UIAs	Unruptured intracranial aneurysms
MP	Morphological parameter
CFD	Computational fluid dynamics
WSS	Wall shear stress
MCA	Middle cerebral artery
CTA	Computer Tomography angiogram
MRI	Magnetic resonance imaging
